# Neuromodulation in Pediatric Epilepsy: When All Is Not About Resection—A Review

**DOI:** 10.1177/15357597261419178

**Published:** 2026-02-26

**Authors:** Juan Toro Perez, Kathryn Lalor, Monika Jones, Ahmed Abdelmoity, Kimberly Houck, Aria Fallah, Saadi Ghatan, Syed Anwar, Eisha Christian, Daniel San Juan Orta, Shilpa Reddy, Inna Hughes, Sarah A. Kelley

**Affiliations:** 1 27338 Children's Hospital of Eastern Ontario, University of Ottawa, Ottawa, Ontario, Canada; 2 Children's of Alabama, 14372 University of Alabama at Birmingham Medical Center, Birmingham, Alabama, USA; 3 Pediatric Epilepsy Surgery Alliance, Los Angeles, California, USA; 4 4204 Children's Mercy, University of Missouri University Hospital, Kansas City, Missouri, USA; 5 3984 Texas Children's Hospital, Baylor College of Medicine, Houston, Texas, USA; 6 21785 Mattel Children's Hospital, University of California Los Angeles Medical Center, Los Angeles, California, USA; 7 Department of Neurosurgery, Icahn School of Medicine at Mount Sinai, New York, USA; 8 8404 Children's National Medical Center, George Washington University Hospital, Washington D.C., USA; 9 Hospital for Sick Children, University of Toronto, Toronto, Ontario, Canada; 10 Epilepsy Clinic, 61614 National Institute of Neurology and Neurosurgery, Mexico City, Mexico; 11 Monroe Carroll Jr. Children's Hospital, 12328 Vanderbilt University Medical Center, Nashville, Tennessee, USA; 12 24402 Golisano Children's Hospital, University of Rochester Medical Center, Rochester, New York, USA; 13 588543 Johns Hopkins Hospital, Johns Hopkins University, Baltimore, Maryland, USA

**Keywords:** neuromodulation, pediatric neuromodulation, presurgical evaluation, epilepsy surgery, drug-resistant epilepsy

## Abstract

Nearly 1% of the global population suffers from epilepsy. There are over 65 million people worldwide with epilepsy and approximately 10.5 million of those are children. One third of these people have drug-resistant epilepsy (DRE). Neuromodulation is an adjunct treatment option for these patients. This manuscript aims to describe the history, patient/parent perspective, current use, and future directions of neuromodulation in the management of pediatric DRE. We conducted a nonsystematic search of the literature through different databases as part of the Pediatric State of The Art Symposium at the 2025 American Epilepsy Society meeting in Atlanta. Three neuromodulation therapies, all using implanted devices and electrodes, have been approved by the Food and Drug Administration (FDA) to treat DRE, namely, vagus nerve stimulation (VNS) (1997 and lately 2017), deep brain stimulation of the anterior nucleus of the thalamus (ANT-DBS) in 2018 (2010 in Europe), and responsive neurostimulation (RNS) in 2013. Only VNS is approved in pediatric patients. Emerging noninvasive neuromodulation modalities such as noninvasive VNS and transcranial magnetic stimulation showed mild adverse effects and exhibit the most encouraging seizure and neurocognitive outcomes to date, but larger multicenter studies are essential before these modalities can be offered and integrated into standard pediatric epilepsy care.

## Introduction

Nearly 1% of the global population suffers from epilepsy. There are over 65 million people worldwide who are estimated to have epilepsy, including approximately 10.5 million children.^1,2^ One third of these people have drug-resistant epilepsy (DRE).^3^ Neuromodulation is an adjunct treatment option for these patients. This therapy produces electrical or magnetic stimulation to modify the nerve activity through targeted delivery of stimuli, involving direct or induced electrical current, with the goal of decreasing seizure frequency, severity, and/or duration.^4^ Three neuromodulation therapies, all using implanted devices and electrodes, have been approved by the Food and Drug Administration (FDA) to treat DRE, namely, vagus nerve stimulation (VNS) (1997 and lately 2017), deep brain stimulation of the anterior nucleus of the thalamus (ANT-DBS) in 2018 (2010 in Europe), and responsive neurostimulation (RNS) in 2013.^5^ VNS is currently the only one of these treatment approved by the FDA to pediatric patients with a DRE indication.

At the 2025 American Epilepsy Society Meeting the Pediatric State of the Art Symposium addressed the history, patient/parent perspective, current use, and future directions of neuromodulation in the management of pediatric DRE. This manuscript serves to highlight the up-to-date information presented on this topic.

## Methods

A nonsystematic search of the bibliography was done through different databases including PubMed, Medline, Scopus, and Lillacs as part of the Pediatric State of The Art Symposium at the 2025 American Epilepsy Society meeting in Atlanta.

## History of Neuromodulation in Epilepsy

Neuromodulation first entered the literature in 1884 with the publication of “the galvanic current effect in the pneumogastric nerve” by Leonard Corning using insulated sponge electrodes; he posited that the device reduced blood flow into the central nervous system to create its effect.^6^ Fifty years later, a report stated that stimulation of the vagus nerve (VN) increased the potential of the orbitofrontal cortex (based on cortical electrograms) and decreased blood pressure due to anoxemia.^7^ Subsequently, studies in cats (Encéphale isolé) demonstrated it was possible to reduce or eliminate spontaneous cortical spindles and strychnine induced spikes through repetitive central vagal stimulation.^8^ In the 1990s, human studies with a VNS implantable device demonstrated seizure reduction of more than 50% in patients with focal epilepsy.^9,10^ Other studies done only in the pediatric population reported similar outcomes.^11–13^ Recently, VNS was found to be effective in generalized tonic clonic seizures with 77% seizure reduction in adult and pediatric patients.^14^

Subsequently, Penfield studied neurostimulation by using cortical electrical probes. This formed the foundation for the development of a stereotactic apparatus improving the accuracy of neurosurgical procedures and electrical stimulation (later becoming the deep brain stimulator (DBS)).^15,16^ Initial targets included the brainstem, frontopolar cortex, spinal cord, and thalamus based on the localization of Parkinson disease, psychiatric disorders, and chronic pain.^16–18^ Advances during the 1980s and 1990s led to decreased size of the devices, the addition of lithium batteries increasing longevity.^19^ More recently, a randomized clinical trial (SANTE) demonstrated the efficacy and tolerability of DBS in people with epilepsy (PWE).^20^ This technology has advanced to include real time monitoring of electrical activity and stimulation and subsequently the development of external responsive neurostimulators.^21,22^ Researchers found the electrographic seizures were suppressed by direct brain stimulation.^21,22^ Later advances in automated seizure detection led to the development of RNS.^23^

## Expectations of PWE and Their Families Regarding Neuromodulation

The landscape of pediatric epilepsy surgery today encompasses a growing menu of surgical treatments for pediatric DRE, including neuromodulation. This treatment should be framed as a meaningful and proactive option rather than as a fallback when resective surgery is not recommended. Families and clinicians could discuss minimally invasive procedures as focused ultrasound, endovascular embolic, or robotic thermocoagulative approaches, in addition to VNS, RNS, and DBS neuromodulation technologies.^24,25^

Parent decision-making studies in neuromodulation technologies for DRE identified three branches: features of the intervention; decision drivers shaped by relational trust and contextual barriers; and the sources/quality of information parents use.^26^ Parents are most concerned about the direct risks and benefits of surgical and device-based interventions. There is a general aversion to open or highly invasive surgical procedures, and parents often prefer to try less invasive neurotechnologies first. In doing so they underappreciate the risks of ongoing seizures, instead focusing on the risks of new procedures and technologies. Parents weigh their child's overall quality of life improvements (including cognition, behavior, and independence) at least as heavily as seizure freedom. Barriers such as access to treatment, financial challenges, insurance limitations, and family considerations are equally influential. Parents obtain decision-making information from a variety of sources, including clinical teams, online materials, peer networks, and social media.^27,28^

The term “palliative” is commonly used to describe neuromodulation technologies, yet as Englot notes “… we rarely refer to anti-seizure medications (ASM) as ‘palliative,’ even though they may be considered such. So, why would we use the word when referring to epilepsy surgery?”.^29^ This terminology undervalues significant life-changing benefits to cognition, safety, and daily function even when seizures do not fully resolve. Curative surgeries do not guarantee seizure freedom forever.^30^ For many in the general public, “palliative” connotes pain management, end-of-life care, and the notion of “giving up.”^31^ Recent commentary supports retiring “palliative” from epilepsy surgery altogether, in favor of language that reflects the comprehensive improvements these interventions – including neuromodulation technologies – can offer.^32^

Parents of children with DRE experience significantly elevated levels of stress even before surgery, driven by uncertainty, constant vigilance, and the demanding nature of care.^33^ Pediatric neurosurgery adds an undeniable layer of stress for families, with evidence showing that almost half of parents display enough symptoms to warrant a diagnosis of post-traumatic stress disorder (PTSD) following the child's neurosurgical procedure.^34^ Studies demonstrate that while stress may decrease postsurgery, levels remain elevated compared to controls.^35^ Because neuromodulation does not promise seizure freedom, clinicians should presume high parental stress will persist, regardless of the intervention's invasiveness.^34^ Notably, mothers suffer disproportionately high rates of depression and anxiety for years after a child's epilepsy surgery.^36^ The pediatric epilepsy surgery journey must be recognized as a source of trauma affecting the entire family that demands a trauma-informed approach throughout both the pre- and postsurgical care of these families to address this persistent psychological burden.

## Choosing the Appropriate Device in Children with DRE Not Amenable to Surgical Resection

### Role of the Vagus Nerve Stimulator

VNS is an FDA-approved as adjunctive therapy for pediatric patients with DRE who are > 4-years-old. This device involves unique multi-level mechanisms including modulation of neurotransmitter expression, cerebral blood flow, and EEG desynchronization. VNS increases acetyl choline,^37^ norepinephrine,^38^ dopamine and GABA,^39^ reduces aspartate and modulates glutamate^39^ balancing inhibition/excitation, influencing synapsis and brain-gut communication.^40–42^ It also reduces inflammation,^37^ increases serotonin and thalamo-cortical blood flow,^43^ as well as desynchronizing EEG rhythms through the amygdala into the limbic system.^44^

In a retrospective cohort of 400 pediatric patients followed over 20 years, VNS demonstrated robust overall responder rates: 44.9% at 3 months, 68.8% at 6 months, 80.9% at 12 months, 85.3% at 24 months, and 90.5% at last follow-up, with 20.5% of patients achieving complete seizure freedom at their final assessment. Early VNS implantation and individualized adjustment of parameters were critical predictors of optimal outcomes. Increasingly higher output and duty cycle parameters are used sooner to improve outcome.^45^ Most adverse events were manageable with protocol adjustments, and quality of life measures improved significantly across multiple domains. Outcomes varied by epilepsy type as seen in Table 1.^45^

**Table 1.** Epilepsy Outcomes of Retrospective Review Following VNS Implantation (Mean Follow Up 4.92 Years) Separated by Epilepsy classification.^45^

Epilepsy type	Outcome
Focal	90.7% responder rate at last follow-up 24.2% achieved seizure freedom
Combined focal and generalized	89.2% responder rate 18.1% seizure freedom
Generalized	88.6% responder rate 17.2% seizure freedom
Lennox Gastaut syndrome	86.7% responder rate 12.5% seizure freedom

### Role of the Deep Brain Stimulator

DBS-ANT is FDA-approved as adjunctive therapy in adults (≥18 years-old) with drug-resistant focal epilepsy. Through off-label use for pediatric epilepsy, DBS has been shown to be safe and effective in pediatric patients with DRE, regardless of epilepsy type or targeted thalamic nuclei, with responder rates varying from 60% to 75% in observational studies.^46–49^ The most common thalamic targets and corresponding epilepsy types based on thalamo-cortical connectivity are included in Table 2.

**Table 2.** Most Common Thalamic Targets for DBS and the Corresponding Localization of Targeted Seizures.^46–49^

Thalamic nucleus	Seizure localization
Anterior (ANT)	Mesial temporal Frontal
Centromedian (CM)	Frontal Broad neocortical Generalized
Pulvinar	Occipital Parietal Temporal (mesial and neocortical)

In young adults with Lennox-Gastaut Syndrome (LGS), class 1 evidence for centromedian DBS (DBS-CM) has shown 50%–59% relative risk in clinical and electrographic seizures, respectively.^50^ In addition to seizure improvements, other benefits of DBS in children include improvements in quality of life and functional outcomes, such as increased school attendance.^51,52^ Long-term studies in adults demonstrate decreased SUDEP risk.^20^ Surgical complications are low in children, comparable to adult populations, and stimulation is well tolerated.^53^

### Role of Responsive Neurostimulation

Although the RNS System is FDA-approved for adult drug-resistant focal epilepsy, its utilization in the pediatric population has expanded as an off-label therapeutic avenue for children as young as three years of age. The system operates on a dual-mechanism hypothesis: acutely delivering abortive stimulation to disrupt ictal patterns and chronically inducing long-term depression of network excitability to suppress cortical synchronization globally. Real-world preliminary experiences, with combined prospective and retrospective data, indicate that RNS is well tolerated in children, with efficacy profiles matching adult literature; approximately 65% of pediatric patients achieve a ≥50% reduction in seizure frequency over short-term follow-up.^54,55^

A significant evolution in pediatric neuromodulation is the application of corticothalamic and bi-thalamic stimulation to address multifocal or generalized epilepsies that are amenable to neither resection nor ablation. By targeting thalamic hubs, specifically the Anterior Nucleus (ANT) for limbic networks, the Centromedian (CM) nucleus for frontal/generalized networks, and the Pulvinar for posterior quadrant onsets, surgeons can modulate large-scale epileptic networks. Recent small single-center data regarding bilateral CM stimulation has demonstrated robust outcomes, with 60% of patients with LGS and 100% of patients with generalized epilepsy achieving ≥50% seizure reduction in retrospective review.^54^ Notably, early intervention in these developmental epileptic encephalopathies may mitigate maladaptive network reorganization and subsequent cognitive regression.^55^

Surgical implantation in the pediatric cohort requires distinct technical modifications to address anatomical constraints. Adverse effects were predominantly associated with high charge density in monopolar-cathodal configurations and were largely resolved by adjusting stimulation parameters or transitioning to bipolar configurations.^56^ Main characteristics of the neuromodulation devices described above are summarized in Table 3.

**Table 3.** Indication, Mechanisms, Side Effects, Strengths, and Limitations of the Neuromodulation Devices.

Device	Indications (age and seizure type)	Mechanisms	Side effects	Strengths	Limitations
VNS	≥4-year-old focal seizures	Increase norepinephrine reducing inflammation Increase GABA, reduce aspartate, balancing inhibition/excitation Increase serotonin Increase thalamo-cortical blood flow Desynchronize EEG rhythms	Dysphonia Local paresthesia Cough Shortness of breath Possible worsening of obstructive sleep apnea	Least invasive approved neuromodulation device Broad spectrum High efficacy (seizure burden and quality of life improvement, reducing SUDEP rates) High tolerability with temporary, gradually resolving side effects or parameter adjustments to address side effects	Lack of standardized protocol for patient selection, timing of implantation Lack of remote monitoring or programming Side effects as listed
DBS	≥18 years-old epilepsy refractory to >3 ASM, average 6 seizures per month for at least 3 months, ANT targeting only	Modulates thalamo-cortical activity, interrupting seizure propagation and altering epileptic networks	Surgical complications (e.g., infection, wound dehiscence, hardware-related) Potential mood and memory adverse events in trials	Decreased SUDEP risk, improved seizure severity, improved quality of life. No need for data downloads	No RCT evaluating effectiveness Lack of standardized protocol for patient selection, timing of implantation
RNS	Adults. Used off-label in pediatrics (studies cite patients as young as 3 years old). Focal: DRE (up to two foci). Generalized/multifocal: used for regional neocortical, multifocal, or generalized epilepsies (e.g., LGS, absence) via thalamic targeting (ANT, CM, Pulvinar).	Closed-loop/responsive: Continuously monitors ECoG, detects specific ictal patterns, and delivers abortive stimulation to desynchronize pathological activity. Dual mechanism: Acute: immediate disruption of seizure propagation (<1% of stimulations). Chronic: long-term depression (LTD) of network excitability/neuroplasticity (>99% of stimulations).	Surgical/hardware: Infection (approx. 6% in some cohorts). Lead fracture (3%). Scalp erosion. Discomfort at implant site. Stimulation-related: Sensory: paresthesia or painful shocks (often improved by switching to bipolar stimulation). Motor: dystonic posturing, extremity jerking, nystagmus.	Diagnostic value: provides “ECoG at the source,” allowing long-term monitoring of seizure burden, medication response, and seizure cycles. Efficacy: high responder rates in pediatrics (∼60%–62% achieve ≥50% reduction) Longevity: battery life ∼11.5 years	Operational burden: requires regular data upload by patient and family. Growth: skull growth can cause lead pullout (requires leaving generous lead slack) Scalp: Thin scalp increases erosion risk Imaging: More MRI artifact than DBS.

## Artificial Intelligence (AI) and Machine Learning for sEEG Seizure Detection, Localization, and to Predict Seizure Freedom

AI, particularly through graph-based deep learning frameworks, has the potential to significantly impact neuromodulation and epilepsy surgery by decoding complex neural network interactions that underline seizure generation and propagation. Graph Neural Networks (GNNs) model the intricate thalamo-cortical connectivity responsible for epileptic activity. Utilizing stereo electroencephalography (sEEG) to represent each electrode channel as a node and inter channel connectivity as weighted edges, GNN captures both spatial and temporal dependencies of brain dynamics.^57^ Spatiotemporal GNNs models and dual task architectures integrate high resolution electrophysiological recordings with imaging modalities like MRI to attempt to predict surgical outcomes and localize seizure onset zones with high accuracy (> 90% in seizure wise validation). They may also reveal key biomarkers of poor outcomes, including excessive thalamo-cortical synchronization and dense network topologies at seizure onset.^58^

Combining multimodal neuroimaging and electrophysiological data, AI is emerging as a potential tool capable of better identifying patients and cerebral targets for neuromodulation using precision neuronal network mapping.^57,58^

## Global Perspectives: Epilepsy Surgery in Limited Resource Settings

Of the 65 million individuals worldwide with active epilepsy, close to 80% are in low-middle income countries (LMIC), where the burden is disproportionately higher. Yet, specialized treatment services remain scarce, and much of the population has no access to advanced diagnostics or surgical intervention.^59–62^

There is a large gap in access to epilepsy surgery, which is complex, resource-intensive, and dependent on advanced diagnostic tools such as video-EEG monitoring, MRI, PET, MEG in addition to neurosurgical tools which include navigation systems, microscopes, robotic systems, ablation systems, and neuromodulatory implants. In addition to technology, successful epilepsy surgery requires a coordinated multidisciplinary team including epileptologists, neurosurgeons, neurophysiologists, anesthesiologists, and specialized nursing support. These capacities are the exception in LMICs, where even basic EEG, cross sectional imaging, trained personnel, and infrastructure may be limited which reinforces global inequities in epilepsy outcomes.^60^

Ukraine is discussed as a detailed example of how a middle-income country can progressively build epilepsy surgery capability. A bidirectional collaborative partnership between the Hospital for Sick Children in Toronto and multiple Ukrainian centers began in 2012, with the goal of expanding pediatric surgical and clinical expertise through hands-on observerships, clinical fellowships, and advisory training.^63,64^

More than 60 Ukrainian physicians and surgeons have travelled to Toronto for month-long observerships through this initiative, participating in clinical duties and integration of research into clinical activity. These experiences had previously been inaccessible in Ukraine. Conversely, Canadian specialists travel to Ukraine to provide clinical and surgical guidance in areas such as pediatric neurosurgery, neonatology, and neurology.^64^

With ongoing efforts, they are achieving outcomes comparable to high-income countries in selected epilepsy disorders and moving toward advanced techniques such as sEEG and ablation. The Ukrainian model underscores the value of long term, stepwise capacity building supported by international collaboration. It demonstrates that meaningful progress toward specialized epilepsy surgery is possible even where initial resources are limited.^65–68^

## Future of Neuromodulation: Emerging Technologies in Neuromodulation

Emerging noninvasive and minimally invasive neuromodulation management options are expanding in pediatric epilepsy, offering alternatives to pharmacologic, dietary, and current surgical approaches. Several novel stimulation modalities including transcranial magnetic stimulation (TMS), tDCS, noninvasive vagus nerve stimulation (nVNS), electroconvulsive therapy (ECT), focused ultrasound stimulation (FUS), and chronic subthreshold cortical stimulation (CSCS) are discussed below regarding current evidence, mechanisms, and limitations.

### Role of the Cathodal Transcranial Direct Current Stimulation in Pediatric Epilepsy

Transcranial direct current stimulation (tDCS) is a noninvasive, safe, cost-effective, and easy to apply technique, that modulates cortical excitability through subthreshold membrane depolarization or hyperpolarization.^69,70^ The effects of a single 20–30-min session in humans can persist for up to 90 min.^71^ This device remains investigational and is not yet approved by the FDA for clinical use.

The mechanism of tDCS is partially understood. Cathodal tDCs (c-tDCs) decrease the neuronal hyperexcitability associated with epilepsy.^72^ The c-tDCs mechanisms include shifting neuronal membrane potential towards hyperpolarization, reducing synaptic transmission particularly mediated by N-methyl-D-aspartate receptors and potentially inducing long-term depression (LTD)-like effects with transmembrane protein migraine, cell migration of neurons and glial cells, and anti-inflammatory effects.^72,73^

Cathodal tDCS has been used in heterogeneous pediatric and adult clinical patients suffering focal refractory epilepsy such as Rasmussen Syndrome or Infantile Epileptic Spasms with promising results.^72,74^ Recent randomized clinical trials have demonstrated frequency seizure reduction of 40%–50%, with only minor, self-limited adverse effects.^75–77^

This emerging technique appears feasible for implementation in pediatric patients with limited resources and may serve as an adjunctive, home-based therapy.^78^ Moreover, it carries the potential to enhance cognitive functions frequently compromised by epilepsy and its associated neuropsychiatric comorbidities.^79^

### Role of the Noninvasive VNS in Pediatric Epilepsy

Noninvasive VNS is represented by transcutaneous VNS, transcutaneous cervical VNS (tcVNS), percutaneous auricular VNS (paVNS), and transcutaneous auricular VNS (taVNS). These therapies target the afferent vagus nerve fibers beyond the nucleus of the solitary tract which integrates not only visceral but also somatic afferents.^80^ These devices have shown responder rates (> 50% seizure reduction) ranging from 40% to 57% with safety profiles superior to implanted VNS. Adverse side effects were mostly headache, ear pain, and skin alteration and rated as mild to moderate.^81^

A prospective pilot trial in 14 children with epilepsy demonstrated efficacy with seven of 13 children being responders and four of 13 children being seizure free after 4 months and persisting after 6 months of stimulation. Reduction of seizure frequency was significant compared to baseline after 4 and 6 months of stimulation (*p* < 0.05). Tolerability was generally good, two children experienced mild skin ulceration.^82,83^

### Role of the Electroconvulsive Therapy

ECT, traditionally reserved for psychiatric conditions, has demonstrated efficacy in refractory status epilepticus as described in case reports, though relapse rates were high and standardized pediatric protocols are lacking.^83,84^

### Role of the Focused Ultrasound Stimulation

FUS represents a preclinical modality capable of focal, nonthermal neuromodulation, with early human studies suggesting variable seizure outcomes but highlighting safety concerns including transient cognitive effects. Low intensity focused ultrasound (LIFU) can modulate neuronal activity and could be used to lower cortical neuronal hyper-excitability in epilepsy patients in a noninvasive manner.^83,85^

### Role of the Chronic Subthreshold Cortical Stimulation

CSCS, an open-loop stimulation via subdural electrodes, targets the location of multifocal or eloquent seizure onset, has shown >90% responder rates and sustained seizure reduction in small cohorts, suggesting promise for multifocal epilepsies or for epileptogenic foci in eloquent cortex.^83,86^

## Making the Neuromodulation Modality Decision in Pediatric Epilepsy

A patient with DRE, who is not a candidate for resection/ablation or disconnection, should be considered for neuromodulation. Typical examples are patients with generalized DRE, focal DRE with a nonlocalized epileptogenic zone, focal DRE with localized epileptogenic zone in an eloquent region, or focal DRE with two or more epileptogenic foci.^87,88^ At present, there is limited evidence comparing different modalities of neurostimulation to guide formation of a formal algorithm. A meta-analysis conducted in 2022 looked at 30 studies, only six of which were randomized controlled trials and none of which included head to head comparisons of different modalities.^89^ When long-term outcomes data were pooled from all studies of each device the results overall suggested that RNS and DBS are more effective at decreasing seizure frequency than VNS. However, length of follow up was shorter in the VNS studies and patient selection varied.

Given the lack of direct comparison trials and difficulties in comparing studies of individual devices, at this time providers should approach consideration of neurostimulation with a patient and family-centered approach. Known potential risks and benefits for each device can be discussed in relation to an individual patient's presentation, keeping in mind specific epilepsy type, socio-economic and geographic factors, and comorbidities. For example, VNS requires the least amount of maintenance and is typically the lowest cost option with RNS currently requiring the most in-person programming and maintenance. For people living far from surgical epilepsy centers or have higher cost sharing insurance plans, these factors may weigh more heavily in the decision making process. Alternatively, VNS may also pose higher risk for irritation from the sensation of stimulation and/or device placement location for certain young people and also has less data regarding long-term seizure reduction.^87^ For PWE who have a high burden of epilepsy and may have comorbidities such as autism with high sensory sensitivity, other modalities may be considered as a first option.

Because RCTs may be difficult or even impossible to conduct to directly compare these neuromodulation modalities, an emphasis should be placed on comprehensive registries, including outcome data, so that cohorts of similar patients can be more directly compared.

## Conclusion

As demonstrated at the 2025 American Epilepsy Society Meeting the Pediatric State of the Art Symposium, neurostimulation is a viable, active treatment that should be considered early on in the pediatric population with DRE. It has real potential to make impactful changes on the burden of epilepsy. In addition to reducing seizure frequency and severity, these therapies have the potential to positively affect quality of life and cognition as these therapies improve seizures and allow for decreased medication burden and side effects. More data needs to be gathered to help guide clinician teams and families making timing and modality decisions regarding neurostimulation. Until then, providers should have an informed discussion regarding potential benefits and risks for each individual patient to help determine recommendations and the ultimate decision to move forward with a specific therapy.

## References

[1] Moshé SL Perucca E Ryvlin P Tomson T . Epilepsy: New advances. Lancet. 2015;385(9971):884‐898. doi:10.1016/S0140-6736(14)60456-6 25260236

[2] Guerrini R . Epilepsy in children. Lancet. 2006;367(9509):499‐524. doi:10.1016/S0140-6736(06)68182-8 16473127

[3] Fisher RS van Emde Boas W Blume W , et al. Epileptic seizures and epilepsy: definitions proposed by the international league against epilepsy (ILAE) and the international bureau for epilepsy (IBE). Epilepsia. 2005;46(4):470‐472. doi:10.1111/j.0013-9580.2005.66104.x 15816939

[4] Hachem LD Yan H Ibrahim GM . Invasive neuromodulation for the treatment of pediatric epilepsy. Neurotherapeutics. 2019;16(1):128‐133. doi:10.1007/s13311-018-00685-1 30378003 PMC6361060

[5] Simpson HD Schulze-Bonhage A Cascino GD , et al. Practical considerations in epilepsy neurostimulation. Epilepsia. 2022;63(10):2445‐2460. doi:10.1111/epi.17329 35700144 PMC9888395

[6] Corning JL . Electrization of the sympathetic and pneumogastric nerves, with simultaneous bilateral compression of the carotids. New York Med. J. 1984;39:212‐2156. http://resource.nlm.nih.gov/101659668 .

[7] Bailey P Bremer F . A sensory cortical representation of the vagus nerve: with a note on the effects of low blood pressure on the cortical electrogram. J. Neurophysiol. 1938;1(5):1405‐1412. doi:10.1152/jn.1938.1.5.405

[8] Zanchetti A Wang SC Moruzzi G . The effect of vagal afferent stimulation on the EEG pattern of the cat. Electroencephalogr Clin Neurophysiol. 1952 Aug;4(3):357‐361. doi:10.1016/0013-4694(52)90064-3. PMID: 12989094. 12989094

[9] Penry JK Dean JC . Prevention of intractable partial seizures by intermittent vagal stimulation in humans: preliminary results. Epilepsia. 1990;31(2):S40‐S43. doi:10.1111/j.1528-1157.1990.tb05848.x 2121469

[10] Uthman BM Wilder BJ Penry JK , et al. Treatment of epilepsy by stimulation of the vagus nerve. Neurology. 1993;43(7):1338‐1345. doi:10.1212/wnl.43.7.1338 8327135

[11] Murphy JV Hornig G Schallert G . Left vagal nerve stimulation in children with refractory epilepsy. Preliminary Observations. Arch Neurol. 1995;52(9):886‐889. doi:10.1001/archneur.1995.00540330064016 7661726

[12] Schallert G Murphy J Hornig G Tilton C . Vagus nerve stimulator: Experience in 20 children with refractory epilepsies. Epilepsia. 1996;37:119.

[13] Lundgren J Amark P Blennow G Strömblad LG Wallstedt L . Vagus nerve stimulation in 16 children with refractory epilepsy. Epilepsia. 1998;39(8):809‐813. doi:10.1111/j.1528-1157.1998.tb01173.x 9701369

[14] Suller Marti A Verner R Keezer M , et al. Reduction of generalized tonic-clonic seizures following vagus nerve stimulation therapy: CORE-VNS study 24-month follow-up. Epilepsia. 2025;66(7):2307‐2314. doi:10.1111/epi.18371 40167365 PMC12291022

[15] Penfield W . Epilepsy and surgical therapy. Arch Neurol Psychiatry. 1936;36(3):449‐484. doi:10.1001/archneurpsyc.1936.02260090002001

[16] Spiegel EA Wycis HT Marks M Lee AJ . Stereotaxic apparatus for operations on the human brain. Science. 1947;106(2754):349‐350. doi:10.1126/science.106.2754.349 17777432

[17] Gildenberg PL . History of electrical neuromodulation for chronic pain. Pain Medicine. 2006;7(s1):S7‐S13. doi:10.1111/j.1526-4637.2006.00118.x

[18] Hosobuchi Y Adams JE Rutkin B . Chronic thalamic stimulation for the control of facial anesthesia dolorosa. Arch Neurol. 1973;29(3):158‐161. doi:10.1001/archneur.1973.00490270040005 4591720

[19] Gardner J . A history of deep brain stimulation: Technological innovation and the role of clinical assessment tools. Soc Stud Sci. 2013;43(5):707‐728. doi:10.1177/0306312713483678. PMCID.

[20] Salanova V Witt T Worth R , et al. Long-term efficacy and safety of thalamic stimulation for drug-resistant partial epilepsy. Neurology. 2015;84(10):1017‐1025. doi:10.1212/WNL.0000000000001334 25663221 PMC4352097

[21] Inaji M Yamamoto T Kawai K Maehara T Doyle WK . Responsive neurostimulation as a novel palliative option in epilepsy surgery. Neurol Med Chir (Tokyo). 2021;61(1):1‐11. doi:10.2176/nmc.st.2020-0172 33268657 PMC7812309

[22] Kossoff EH Ritzl EK Politsky JM , et al. Effect of an external responsive neurostimulator on seizures and electrographic discharges during subdural electrode monitoring. Epilepsia. 2004;45(12):1560‐1567. doi:10.1111/j.0013-9580.2004.26104.x 15571514

[23] Peters TE Bhavaraju NC Frei MG Osorio I . Network system for automated seizure detection and contingent delivery of therapy. J Clin Neurophysiol. 2001;18(6):545‐549. doi:10.1097/00004691-200111000-00004 11779967

[24] Xu R Srinivasan E Hung A . Endovascular embolization for medically refractory pediatric epilepsy: a case series. J Neurointerv Surg. Published online May 30, 2025. doi:10.1136/jnis-2025-023265 40447330

[25] Lescrauwaet E Vonck K Sprengers M , et al. Recent advances in the use of focused ultrasound as a treatment for epilepsy. Front. Neurosci. 2022;16:886584. doi:10.3389/fnins.2022.886584 35794951 PMC9251412

[26] Hrincu V McDonald PJ Connolly MB , et al. Parent decision making for neurotechnologies for pediatric drug-resistant epilepsy. J Child Neurol. 2021;36(11):943‐949. doi:10.1177/08830738211015010 34078159 PMC8458226

[27] Hatoum R Nathoo-Khedri N Shlobin NA Wang A Weil AG Fallah A . Barriers to epilepsy surgery in pediatric patients: a scoping review. Seizure. 2022;102:83‐95. doi:10.1016/j.seizure.2022.08.013 36209677

[28] Cohen NT Oluigbo CO Gaillard WD . Breaking barriers to pediatric epilepsy surgery utilization. J Pediatr. 2025;276:114283. doi:10.1016/j.jpeds.2024.114283 39216618

[29] Englot DJ . Epilepsy surgery is not palliative. Epilepsia. 2024;65(2):281‐282. doi:10.1111/epi.17815 37914404 PMC12342346

[30] Bellamkonda N Phillips HW Chen JS , et al. Epilepsy surgery for Rasmussen encephalitis: the UCLA experience. J Neurosurg Pediatr. 2020;26(4):389‐397. doi:10.3171/2020.4.PEDS2098 32679562

[31] Fliedner MC Zambrano SC Eychmueller S . Public perception of palliative care: a survey of the general population. Palliat Care Soc Pract. 2021;8(15):26323524211017546. doi:10.1177/26323524211017546 PMC8191057 34164622

[32] Zhong C Yang K Wang N , et al. Advancements in surgical therapies for drug-resistant epilepsy: a paradigm shift towards precision care. Neurol Ther. 2025;14:467‐490. doi: 10.1007/s40120-025-00710-4 39928287 PMC11906941

[33] Operto FF Pastorino Grazia MG Pippa F , et al. Psychiatric symptoms and parental stress in children and adolescents with epilepsy. Front Neurol. 2021;12:794379. doi:10.3389/fneur.2021.794379 PMC8694379 34956058

[34] Beaudoin W Moore H Bliss L Souster J Mehta V . Prevalence of post-traumatic stress disorder in caregivers of pediatric neurosurgical patients. Childs Nerv Syst. 2021;37(3):959‐967. doi:10.1007/s00381-020-04938-3 33111174

[35] Braams O Meekes J Braun KPJ , et al. Parenting stress does not normalize after child's epilepsy surgery. Epilepsy Behav. 2015;42:147‐152. doi:10.1016/j.yebeh.2014.10.034 25468727

[36] Smith ML Puka K Speechley KN , et al. Trajectories of parent well-being in children with drug-resistant epilepsy. Epilepsia. 2023;64(12):3342‐3353. doi:10.1111/epi.17797 37828819

[37] Bonaz B Picq C Sinniger V Mayol JF Clarençon D . Vagus nerve stimulation: from epilepsy to the cholinergic anti-inflammatory pathway. Neurogastroenterol Motil. 2013;25(3):208‐221. doi:10.1111/nmo.12076 23360102

[38] Follesa P Biggio F Gorini G , et al.. Vagus nerve stimulation increases norepinephrine concentration and the gene expression of BDNF and bFGF in the rat brain. Brain Res. 2007;1179(7):28‐34. doi:10.1016/j.brainres.2007.08.045 17920573

[39] Walker BR Easton A Gale K . Regulation of limbic motor seizures by GABA and glutamate transmission in nucleus tractus solitarius. Epilepsia. 1999;40(8):1051‐1057. doi:10.1111/j.1528-1157.1999.tb00818.x 10448815

[40] O'Keane V Dinan TG Scott L Corcoran C . Changes in hypothalamic-pituitary-adrenal axis measures after vagus nerve stimulation therapy in chronic depression. Biol Psychiatry. 2005;58(12):963‐968. doi:10.1016/j.biopsych.2005.04.049 16005439

[41] Henry TR . Therapeutic mechanisms of vagus nerve stimulation. Neurology. 2002;59(6 Suppl 4):S3‐14. doi:10.1212/wnl.59.6_suppl_4.s3 12270962

[42] Fuster JM . The prefrontal cortex–an update: time is of the essence. Neuron. 2001;30(2):319‐333. doi:10.1016/s0896-6273(01)00285-9 11394996

[43] Liu WC Mosier K Kalnin AJ Marks D . BOLD fMRI activation induced by vagus nerve stimulation in seizure patients. J Neurol Neurosurg Psychiatry. 2003;74(6):811‐813. doi:10.1136/jnnp.74.6.811 12754361 PMC1738494

[44] Fraschini M Puligheddu M Demuru M , et al.. VNS Induced desynchronization in gamma bands correlates with positive clinical outcome in temporal lobe pharmacoresistant epilepsy. Neurosci Lett. 2013;536(1):14‐18. doi:10.1016/j.neulet.2012.12.044 23333601

[45] Bansal L Jaafar F Kapoor A , et al. Transforming pediatric epilepsy care: real-world insights from a leading single-center study on vagus nerve treatment outcomes. Epilepsia. 2025 Nov 11. doi:10.1111/epi.70002. Epub ahead of print. PMID: 41217293. 41217293

[46] Yan H Toyota E Anderson M , et al. A systematic review of deep brain stimulation for the treatment of drug-resistant epilepsy in childhood. J Neurosurg Pediatr. 2019;23(3):274‐284. doi:10.3171/2018.9.PEDS18417 30544364

[47] Khan M Paktiawal J Piper RJ Chari A Tisdall MM . Intracranial neuromodulation with deep brain stimulation and responsive neurostimulation in children with drug-resistant epilepsy: a systematic review. J Neurosurg Pediatr. 2021;29(2):208‐217. doi:10.3171/2021.8.PEDS21201 34678764

[48] Sharma A Parfyonov M Tiefenbach J , et al. Predictors of therapeutic response following thalamic neuromodulation for drug-resistant pediatric epilepsy: a systematic review and individual patient data meta-analysis. Epilepsia. 2024;65(3):542‐555. doi:10.1111/epi.17883 38265348

[49] Dhaliwal JK Ruiz-Perez M Chari A Piper RJ Tisdall MM Hart M . Deep brain stimulation for epilepsy: a systematic review and meta-analysis of randomized and non-randomized studies of thalamic targeting. Epilepsy Res. 2025;216:107607. doi:10.1016/j.eplepsyres.2025.107607 40516441

[50] Dalic LJ Warren AEL Bulluss KJ , et al. DBS of thalamic centromedian nucleus for lennox-gastaut syndrome (ESTEL trial). Ann Neurol. 2022;91(2):253‐267. doi:10.1002/ana.26280. Erratum in: Ann Neurol. 2022 Aug;92(2):347. doi:10.1002/ana.26356. PMID: 34877694. 34877694

[51] Suresh H Mithani K Warsi N , et al. Add-on deep brain stimulation versus continued vagus nerve stimulation for childhood epilepsy (ADVANCE): a partially randomized patient preference trial. Ann Neurol. 2024;96(2):405‐411. doi:10.1002/ana.26956 38822686

[52] Mithani K Niazi F Suresh H , et al. Deep brain stimulation of the centromedian nucleus for drug-resistant epilepsy in children: quality-of-life and functional outcomes from the CHILD-DBS registry. Epilepsia. 2025 Jul;66(7):2225‐2238. doi:10.1111/epi.18393 40167366 PMC12291001

[53] Balachandar A Verhey LH Mithani K , et al. Surgical complications of deep brain stimulation in children across targets and indications: multicenter analysis of the CHILD-DBS registry. Neurology. 2025;105(9):e214201. doi:10.1212/WNL.0000000000214201 41071966

[54] Nagahama Y Zervos TM Murata KK , et al. Real-World preliminary experience with responsive neurostimulation in pediatric epilepsy: a multicenter retrospective observational study. Neurosurgery. 2021;89(6):997‐1004. doi:10.1093/neuros/nyab343 34528103 PMC8637802

[55] Singh RK Eschbach K Samanta D , et al. Responsive neurostimulation in drug-resistant pediatric epilepsy: findings from the epilepsy surgery subgroup of the pediatric epilepsy research consortium. Pediatr Neurol. 2023;143:106‐112. doi:10.1016/j.pediatrneurol.2023.03.001 37084698

[56] Ahn S Edmonds B Rajaraman RR , et al. Bilateral centromedian nucleus of thalamus responsive neurostimulation for pediatric-onset drug-resistant epilepsy. Epilepsia. 2024;65(8):2131‐2143. doi:10.1111/epi.18031 PMC11315622 38845459

[57] Amir SA Agaronyan A Linguraru MG Oluigbo C Anwar S . Graph-based deep learning for predicting seizure outcome in epilepsy patients with thalamic SEEG contacts. IEEE-EMBS International Conference on Biomedical and Health Informatics. 2025;15(48):1‐6.

[58] Haronyan AA Amir SA Wittayanakorn N Linguraru MG Oluigbo C Anwar SM . Multi-modal graph-based machine learning for predicting surgical outcome in epilepsy patients. In: International Conference on Medical Image Computing and Computer-Assisted Intervention. Springer Nature Switzerland; 2025, September:500‐509.

[59] Samia P Hassell J Hudson J , et al. Epilepsy research in Africa: a scoping review by the ILAE pediatric commission research advocacy task force. Epilepsia. 2022;63(9):2225‐2241. doi:10.1111/epi.17321 35729725

[60] Asadi-Pooya AA Damabi NM Fazelian K Moshfeghinia R Niknam N . How to successfully establish an epilepsy care center in resource-limited countries: a scoping systematic review. Seizure. 2023;109:92‐96. doi:10.1016/j.seizure.2023.06.001 37290225

[61] Di Noia S Bonezzi L Accorinti I Bartolini E . Diagnosis and classification of pediatric epilepsy in sub-Saharan Africa: a comprehensive review. J Clin Med. 2024;13(21):6396. doi:10.3390/jcm13216396 39518535 PMC11545903

[62] Biset G Abebaw N Gebeyehu NA Estifanos N Birrie E Tegegne KD . Prevalence, incidence, and trends of epilepsy among children and adolescents in Africa: a systematic review and meta-analysis. BMC Public Health. 2024;24(1):771. doi:10.1186/s12889-024-18236-z 38475724 PMC10935902

[63] Strelko O Valadka AB Tomycz L , et al. History of tumor, spine, and trauma neurosurgery in Ukraine: growth and resilience. J Neurosurg. 2025;143(4):1151‐1159. doi:10.3171/2025.3.JNS25684 40344773

[64] Christian E Romach M Rutka JT . Assistance amidst air alerts and angst: Ukraine unbroken. J Neurosurg. 2023;140(2):600‐603. doi:10.3171/2023.8.JNS231781 37878008

[65] Bäuerle P Schneider U Holtkamp M Gloveli T Dugladze T . Outlines to initiate epilepsy surgery in low- and middle-income countries. J Integr Neurosci. 2022;21(5):134. doi:10.31083/j.jin2105134 36137955

[66] Gaillard WD Jette N Arnold ST , et al. Task force for pediatric epilepsy surgery, commission for pediatrics, and the surgical commission of the international league against epilepsy. Establishing criteria for pediatric epilepsy surgery center levels of care: report from the ILAE pediatric epilepsy surgery task force. Epilepsia. 2020;61(12):2629‐2642. doi:10.1111/epi.16698 33190227

[67] Williamson PD Jobst BC . Epilepsy surgery in developing countries. Epilepsia. 2000;41(Suppl 4):S45‐S50. doi:10.1111/j.1528-1157.2000.tb01546.x 10963478

[68] Watila MM Xiao F Keezer MR , et al. Epilepsy surgery in low- and middle-income countries: a scoping review. Epilepsy Behav. 2019;92:311‐326. doi:10.1016/j.yebeh.2019.01.001 30738248

[69] Nitsche MA Cohen LG Wassermann EM , et al. Transcranial direct current stimulation: state of the art 2008. Brain Stimul. 2008;1(3):206‐223. doi:10.1016/j.brs.2008.06.004 20633386

[70] Nitsche MA Seeber A Frommann K , et al. Modulating parameters of excitability during and after transcranial direct current stimulation of the human motor cortex. J Physiol. 2005;568(Pt 1):291‐303. doi:10.1113/jphysiol.2005.092429 16002441 PMC1474757

[71] Nitsche MA Paulus W . Sustained excitability elevations induced by transcranial DC motor cortex stimulation in humans. Neurology. 2001;57(10):1899‐1901. doi:10.1212/wnl.57.10.1899 11723286

[72] San-Juan D . Cathodal transcranial direct current stimulation in refractory epilepsy: a noninvasive neuromodulation therapy. J Clin Neurophysiol. 2021;38(6):503‐508. doi:10.1097/WNP.0000000000000717 34261114

[73] Chiang CC Chien ME Huang YC , et al. Cathodal weak direct current decreases epileptic excitability with reduced neuronal activity and enhanced delta oscillations. J Physiol. 2021;603(9):2763‐2782. doi:10.1113/JP287969 PMC12072238 40193544

[74] Ghosh S Nagarajan L . Tolerability and effectiveness of cathodal transcranial direct current stimulation in children with refractory epilepsy: a case series. Brain Sci. 2023;13(5):760. doi:10.3390/brainsci13050760 37239232 PMC10216667

[75] Hendi NI AbuSammour Y Khaled M , et al. Efficacy of transcranial direct current stimulation on seizure control in patients with refractory epilepsy: a systematic review and meta-analysis of randomized controlled trials. Neurosurg Rev. 2025;48(1):515. doi:10.1007/s10143-025-03657-0 40533536 PMC12177013

[76] Ding XT Hu MY Wang C , et al. The safety and effectiveness of tDCS for epileptic patients: a systematic review and meta-analysis. Complement Ther Med. 2025;89:103142. doi:10.1016/j.ctim.2025.103142 39909364

[77] Hawas Y Abbas A Alkhawaldeh IM , et al. Efficacy and safety of transcranial direct current stimulation (tDCS) in treatment of refractory epilepsy: an updated systematic review and meta-analysis of randomized sham-controlled trials. Neurol Sci. 2025;46(2):671‐687. doi:10.1007/s10072-024-07866-1 39532798 PMC11772517

[78] Palm U Kumpf U Behler N , et al. Home use, remotely supervised, and remotely controlled transcranial direct current stimulation: a systematic review of the available evidence. Neuromodulation. 2018;21(4):323‐333. doi:10.1111/ner.12686 28913915

[79] Chen Y Ou Z Hao N , et al. Transcranial direct current stimulation in the management of epilepsy: a meta-analysis and systematic review. Front Neurol. 2024;15:1462364. doi:10.3389/fneur.2024.1462364 39588230 PMC11586187

[80] Neuhuber WL Berthoud HR . Functional anatomy of the vagus system - emphasis on the somato-visceral interface. Auton Neurosci. 2021;236:102887. doi:10.1016/j.autneu.2021.102887 34634680 PMC8627476

[81] von Wrede R Surges R . Transcutaneous vagus nerve stimulation in the treatment of drug-resistant epilepsy. Auton Neurosci. 2021 Nov;235:102840. doi:10.1016/j.autneu.2021.102840 34246121

[82] He W Jing X Wang X , et al. Transcutaneous auricular vagus nerve stimulation as a complementary therapy for pediatric epilepsy: a pilot trial. Epilepsy Behav. 2013;28(3):343‐346. doi:10.1016/j.yebeh.2013.02.001 23820114

[83] Starnes K Miller K Wong-Kisiel L Lundstrom BN . A review of neurostimulation for epilepsy in pediatrics. Brain Sci. 2019;9(10):283. doi:10.3390/brainsci9100283 31635298 PMC6826633

[84] Incecik F Horoz OO Herguner OM Yıldızdas D Altunbasak S . Electroconvulsive therapy for refractory status epilepticus in a child: a case report. Ann Indian Acad Neurol. 2015;18(3):364‐365. doi:10.4103/0972-2327.157250 26425029 PMC4564486

[85] Lescrauwaet E Vonck K Sprengers M , et al. Recent advances in the use of focused ultrasound as a treatment for epilepsy. Front Neurosci. 2022;16:886584. doi:10.3389/fnins.2022.886584 35794951 PMC9251412

[86] Lundstrom BN Worrell GA Stead M Van Gompel JJ . Chronic subthreshold cortical stimulation: a therapeutic and potentially restorative therapy for focal epilepsy. Expert Rev. Neurother. 2017;17(7):661‐666. doi:10.1080/14737175.2017.1331129 28532252

[87] Simpson HD Schulze-Bonhage A Cascino GD , et al. Practical considerations in epilepsy neurostimulation. Epilepsia. 2022;63(10):2445‐2460. doi:10.1111/epi.17329 35700144 PMC9888395

[88] Wong S Mani R Danish S . Comparison and selection of current implantable anti-epileptic devices. Neurotherapeutics. 2019;16(2):369‐380. doi:10.1007/s13311-019-00727-2 31062294 PMC6554379

[89] Touma L Dansereau B Chan AY , et al.. Neurostimulation in people with drug-resistant epilepsy: systematic review and meta-analysis from the ILAE surgical therapies commission. Epilepsia. 2022;63(6):1314‐1329. doi: 10.1111/epi.17243 35352349

